# Modelling the role of correctional services on gangs: insights through a mathematical model

**DOI:** 10.1098/rsos.170511

**Published:** 2017-10-11

**Authors:** F. Nyabadza, C. P. Ogbogbo, J. Mushanyu

**Affiliations:** 1 South Africa Center of Epidemiological Modelling and Analysis, Stellenbosch University, Stellenbosch, South Africa; 2 Department of Mathematics and Applied Mathematics, University of Ghana, Accra, Ghana; 3 Department of Mathematics, University of Zimbabwe, Harare, Zimbabwe

**Keywords:** gangs, correctional services, gang reproduction number, numerical simulations

## Abstract

Research has shown that gang membership increases the chances of offending, antisocial behaviour and drug use. Gang membership should be acknowledged as part of crime prevention and policy designs, and when developing interventions and preventative programmes. Correctional services are designed to rehabilitate convicted offenders. We formulate a deterministic mathematical model using nonlinear ordinary differential equations to investigate the role of correctional services on the dynamics of gangs. The recruitment into gang membership is assumed to happen through an imitation process. An epidemic threshold value, $R_{g}$, termed the gang reproduction number, is proposed and defined herein in the gangs’ context. The model is shown to exhibit the phenomenon of backward bifurcation. This means that gangs may persist in the population even if $R_{g}$ is less than one. Sensitivity analysis of $R_{g}$ was performed to determine the relative importance of different parameters in gang initiation. The critical efficacy ε* is evaluated and the implications of having functional correctional services are discussed.

## 1. Introduction

Correctional services in South Africa provide needs-based correctional sentence plans and interventions that are based on an assessment of the security risk and criminal profile of individuals. The corrections target all elements associated with offending behaviour and focus on the offence for which a person was sentenced to correctional supervision, remanded in a correctional centre or released on parole [1]. Correctional programmes and/or interventions can be viewed as a structured set of learning opportunities provided to offenders so they can change for the better and remain crime-free [2]. The assumptions are that offenders have needs that directly cause their criminal behaviour, that these needs can be identified accurately, the apt intervention that will address these needs is available and that this will result in diminished criminal behaviour [3].

On the other hand, gang violence continues to rise and spread in South Africa. Over the past few years, the number of gangs and their activities seems to have increased. By 2005, total number of gangs and gangsters in Cape Town alone was recorded at 130 and 100 000, respectively. In 2013, 12% of 2580 murders in Western Cape province were gang-related [4]. It is reported that a life is lost to gang violence every 5 days on the average in the Cape Flats [5]. In view of this, government and security agents, consider any model to reduce gang and gang activities as crucial and even priceless. Apprehended offenders end up in one of the numerous correctional centres in the country. There are approximately 231 correctional centres which includes prisons. About 25 000 people are released from South Africa prisons and jails each month [6]. It is, therefore, pertinent to examine the role of correctional centres in controlling or curbing gang activities.

Mathematical modelling of gang violence and crimes has been carried out by a number of researchers. In [7], a model that details the stability of gang territories and patterns of between-gang violence was studied using Lotka–Volterra equations. In like manner, a predator–prey model was used to study the interaction of gangs and ordinary individuals. Gang members and criminals are viewed as predators and other individuals as the prey [8]. A modified predator–prey model with transmissible disease in both the predator and prey species is proposed and analysed in [9], with the police as predators and gang members as the prey. An SIR model to analyse recruitment into gangs in a manner reminiscent of spread of infectious disease is given in [10]. An interesting model on the use of reaction–diffusion equations to describe the spread of crimes is given in [11]. Criminal behaviour and violence have been studied as a socially infectious disease, using disease modelling techniques [12,13]. An agent-based model to study street gang rivalries is described in [14]. Other mathematical work in the context of crime, punishment and deterrence has been done using game theoretic models [15–18].

The fear, violence and horror associated with gangs is enormous and calls for serious attention. As government seeks solution to the menace of gangs and gangsterism, we investigate the role of correctional centres in tackling the challenge. In this paper, we present a mathematical model which assesses/examines the role of correctional centres in crime reduction. This paper is arranged as follows: in §2, we formulate and establish the basic properties of the model. The model is analysed for stability in §3. Parameter estimation and sensitivity analysis are given in §4. Numerical results on the behaviour of the model are also presented in this section. In §5, we present the application of the model to a real-life situation and the paper is concluded.

## 2. Model formulation

We consider a population whose size is *N*(*t*), at any time *t*. The population is divided into four disjoint independent classes or compartments based on an individual’s status and risk factors with respect to gang membership. The class *S*_*n*_(*t*) represents individuals not at risk of becoming gang members, *S*_*r*_(*t*) represents individuals at risk of becoming gang members, *G*(*t*) represents gang members and lastly, *C*(*t*) represents those in correctional services. The total population at any time *t* is thus given by

$$N(t)=S_{n}(t)+S_{r}(t)+G(t)+C(t).$$

The general population enter the susceptible population at a rate *Λ*. Among individuals entering the susceptible population, we have that a proportion *p* of these individuals are recruited into the class of susceptible individuals not at risk of joining a gang and the complementary proportion (1−*p*) join susceptible individuals at risk of joining a gang. Therefore, we neglect the possible recruitment of individuals already belonging to gangs. Transition rate from no risk susceptibility into at risk susceptibility is represented by *θ*. Unlike in [10], the change in the risk status is not driven by interacting with gang members but simply by change of one’s environmental conditions. A typical example is that of a slowing down economy that results into retrenchment. Once the economic status of an individual changes, then susceptibility to committing crimes may increase. This is particularly important in the South African context as different living environments often determine the risk.

**Figure 1.**
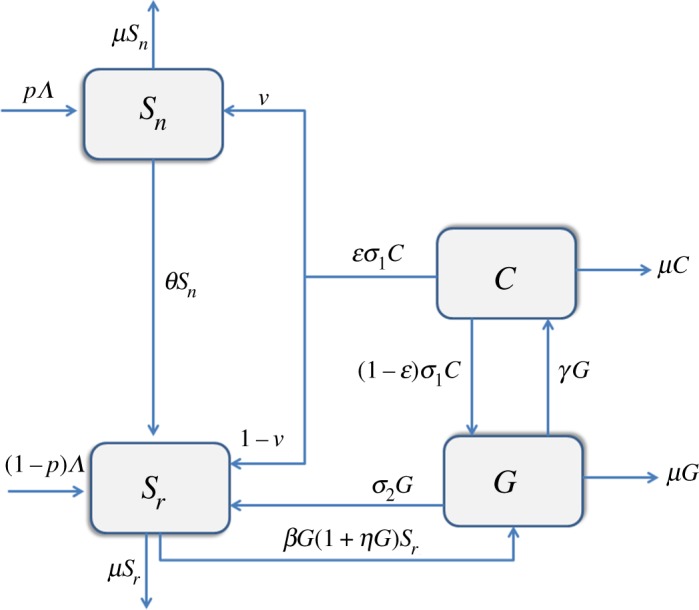
A schematic diagram for the model.

The recruitment of individuals into gangs is assumed to follow an imitation process, described comprehensively in [19]. We propose an initiation function *f*(*S*_*r*_,*G*)=*βG*(1+*ηG*)*S*_*r*_ into gang membership that is driven by imitation, with *β* as the effective contact rate and *η* as the imitation coefficient. Once initiated, we also assume that gang members can either revert back to compartment *S*_*r*_ at a rate *σ*_2_ or are sent to correctional facilities through convictions and sentencing at a rate *γ*. Depending on the efficacy *ε* (where *ε*∈[0,1]) of correctional services, a released inmate may either join a gang again at a rate (1−*ε*)*σ*_1_ or may rejoin the community as either a susceptible at risk at a rate (1−*ν*)*εσ*_1_ or those not at risk at a rate *νεσ*_1_. By efficacy, we mean the measure to which a policy, programme or initiative meets its intended result with *ε*=1 signifying that no individuals will revert to gangs when they leave correctional services. This represents completely effective correctional programmes. *ε*=0 signifies that all individuals in correctional facilities will revert back to gangs upon their release, while 0<*ε*<1 implies that correctional programmes will be effective to some degree. In reality, *ε*∈(0,1). A summary of the description of parameters together with their estimated values is given in table 1. Figure 1 shows the movement of humans as their status with respect to gang membership changes. Combining the parameters, assumptions and the schematic diagram, we have the following general set of nonlinear ordinary differential equations:

2.1 $$\begin{array}{rl} \frac{dS_{n}}{dt} & =p\Lambda +\nu \varepsilon \sigma_{1}C-(\mu +\theta )S_{n},\\ \frac{dS_{r}}{dt} & =(1-p)\Lambda +\theta S_{n}+\sigma_{2}G+(1-\nu )\varepsilon \sigma_{1}C-\beta G(1+\eta G)S_{r}-\mu S_{r},\\ \frac{dG}{dt} & =\beta G(1+\eta G)S_{r}+(1-\varepsilon )\sigma_{1}C-(\mu +\sigma_{2}+\gamma )G\\ \;\text{and}\;\frac{dC}{dt} & =\gamma G-(\mu +\sigma_{1})C, \end{array}\}$$

with the initial conditions:

$$S_{n}(0)=S_{n0}>0,\;S_{r}(0)=S_{r0}>0,\;G(0)=G_{0}\geq 0,\;C(0)=C_{0}\geq 0,$$

where we assume that all the model parameters are positive. The positivity of the solutions of system (2.1) can easily be established if *S*_*n*0_≥0, *S*_*r*0_≥0, *G*_0_≥0, *C*_0_≥0, see for instance [20–22].

**Table 1.** Description of parameters and their estimated values.

parameter	description	estimated value
*γ*	sentencing rate	0.8
*θ*	transition rate from *S*_*n*_ to *S*_*r*_	0.3
*β*	effective contact rate	0.01
*η*	imitation coefficient	0.002
*σ*_1_	release rate from correctional services	0.5
*σ*_2_	natural recovery rate	0.5

## 3. Model analysis

### 3.1. Invariant region

It follows from system (2.1) that d*N*/d*t*=*Λ*−*μN*. Then, $\underset{t\to \infty }{\sup}N(t)\leq \Lambda /\mu$. We can thus study (2.1) in following feasible region

$$\Gamma =\left\{(S_{n}(t),S_{r}(t),G(t),C(t))\in R_{+}^{4}|0\leq N(t)\leq \frac{\Lambda }{\mu }\right\},$$

which is positively invariant with respect to system (2.1). This means that our system is well posed and all solutions of system (2.1) with $(S_{n0},S_{r0},G_{0},C_{0})\in R_{+}^{4}$ remain in *Γ* for all *t*>0.

### 3.2. Gang-free equilibrium and the gang reproduction number

The model has a gang-free equilibrium given by

$$G^{0}=(S_{n}^{0},S_{r}^{0},G^{0},C^{0})=\left(\frac{p\Lambda }{\mu +\theta },\frac{\Lambda (\theta +\mu (1-p))}{\mu (\theta +\mu )},0,0\right),$$

a scenario depicting a gang-free state in the community or society. The gang reproduction number $R_{g}$ of the model, is defined herein in the gang membership context as the average number of people that each single gang member will initiate to a gang during his/her membership in a wholly susceptible population. This threshold quantity is analogous to the basic reproduction number in mathematical epidemiology described in [23,24]. Usually, $R_{g}<1$ implies that gangs will decline, whereas $R_{g}>1$ implies that gangs will persist within a community and $R_{g}=1$ requires further investigation. The determination of $R_{g}$ is done using the next generation matrix approach [24]. This method has been explored in many papers [25–29]. Driessche & Watmough [24] describe the following method to determine the reproduction number:

Let *x*=(*x*_1_,*x*_2_,…,*x*_*n*_)^*t*^, with each *x*_*i*_≥0, be the number of individuals in each compartment. Denote *m* to be the number of compartments corresponding to infected individuals where the epidemiological interpretation of the model determines between infected and uninfected compartments. More than one interpretation is possible for some models. Define $X_{s}$ to be the set of all disease free states given by

$$X_{s}=\{x\geq 0\;|\;x_{i}=0,\;i=1,2,\ldots ,m\}.$$

Let $F_{i}(x)$ be the rate of appearance of new infections in compartment *i*, $V_{i}^{+}(x)$ be the rate of transfer of individuals into compartment *i* by all other means, and $V_{i}^{-}(x)$ be the rate of transfer of individuals out of compartment *i*. It is assumed that each function is continuously differentiable at least twice in each variable. Consider the disease transmission model with non-negative initial conditions given by

3.1 $$\frac{dx_{i}}{dt}=f_{i}(x)=F_{i}(x)-V_{i}(x),\;1\leq i\leq n,$$

where $V_{i}=V_{i}^{-}-V_{i}^{+}$. If *x*_0_ is a disease free equilibrium of (3.1) and *f*_*i*_(*x*) satisfies assumptions (A1)–(A5) given in Driessche & Watmough [24], then the reproduction number of (3.1) is the spectral radius of the next generation matrix *FV* ^−1^ where

$$F=\left[\frac{\partial F_{i}(x_{0})}{\partial x_{j}}\right]\;\text{and}\;V=\left[\frac{\partial V_{i}(x_{0})}{\partial x_{j}}\right]\;\text{with}\;1\leq i,\;j\leq m,$$

where *F* is non-negative and *V* is a non-singular *M*-matrix. Using this method we have

$$F=\left[\begin{array}{c} \beta GS_{r}(1+\eta G)\\ 0\\ 0\\ 0 \end{array}\right]\;\text{and}\;V=\left[\begin{array}{c} (\mu +\sigma_{2}+\gamma )G-(1-\varepsilon )\sigma_{1}C\\ (\mu +\sigma_{1})C-\gamma G\\ (\mu +\theta )S_{n}-p\Lambda -\nu \varepsilon \sigma_{1}C\\ \;\beta G(1+\eta G)S_{r}+\mu S_{r}(1-p)-\Lambda -\theta S_{n}-\sigma_{2}G-(1-\nu )\varepsilon \sigma_{1}C \end{array}\right].$$

The gang members’ compartments are *G* and *C*, giving *m*=2. Then

$$F=\left[\begin{array}{cc} \frac{\beta \Lambda (\theta +\mu (1-p))}{\mu (\theta +\mu )} & 0\\ 0 & 0 \end{array}\right]\;\text{and}\;V=\left[\begin{array}{cc} \left(\mu +\sigma_{2}+\gamma \right) & -(1-\varepsilon )\sigma_{1}\\ -\gamma & \left(\mu +\sigma_{1}\right) \end{array}\right]$$

giving

3.2 $$R_{g}=\frac{\beta \Lambda (\mu +\sigma_{1})(\theta +\mu (1-p))}{\mu (\theta +\mu )(\mu (\gamma +\mu +\sigma_{2})+\sigma_{1}(\gamma \varepsilon +\mu +\sigma_{2}))}.$$

### 3.3. Sensitivity analysis

We examine which model parameter has the greatest effect on the value of the gang reproduction number $R_{g}$. Determining these parameters is useful in reducing the recruitment of new gang members given that $R_{g}$ is directly related to gang initiation. Following Chitnis *et al.* [30], we calculate the sensitivity indices of the gang reproduction number $R_{g}$, to the parameters in the model. These indices indicate how sensitive $R_{g}$ is to a change in each parameter, in other words, this tells us how crucial each parameter is to gang initiation. Since there are usually errors in data collection and presumed parameter values, sensitivity analysis is commonly used to determine the robustness of model predictions to parameter values [30]. Sensitivity indices allow us to measure the relative change in a state variable when a parameter changes. The normalized forward sensitivity index (NFSI) of the gang reproduction number $R_{g}$ to a parameter is the relative change in the variable $R_{g}$ to the relative change in a given parameter. A directly proportional normalized sensitivity index indicates that an increase/decrease in the parameter value brings about an increase/decrease, respectively, in the value of $R_{g}$, whereas, an inversely proportional normalized sensitivity index indicates that an increase in the parameter value brings about a decrease in the value of $R_{g}$. When $R_{g}$ is a differentiable function with respect to each of its parameters, then the sensitivity index may be alternatively defined using partial derivatives as follows.

**Definition 3.1**

Let $R_{g}:V\to W$ and $R_{g}\in C^{1}(V)$, where $V,W\subseteq R^{+}$. Then, for every parameter *q*∈*V* , the NFSI of $R_{g}$ is defined as:

3.3 $$\Upsilon_{q}^{R_{g}}=\frac{\partial R_{g}}{\partial q}\times \frac{q}{R_{g}}.$$

Using an explicit formula for $R_{g}$ (3.2) and definition 3.1, the sensitivity indices of $R_{g}$ with respect to each of its parameters are calculated. Recall that *μ* is the natural death rate. Thus, the sensitivity index of $R_{g}$ with respect to *μ* has been omitted because it is clear that increase in this rate is neither ethical nor practical.

$$\begin{array}{rl} \Upsilon_{\beta }^{R_{g}} & =1,\\ \Upsilon_{\Lambda }^{R_{g}} & =1,\\ \Upsilon_{\sigma_{1}}^{R_{g}} & =\frac{\gamma \mu \sigma_{1}(1-\varepsilon )}{\left(\mu +\sigma_{1})(\mu (\gamma +\mu +\sigma_{2})+\sigma_{1}(\gamma \varepsilon +\mu +\sigma_{2})\right)},\\ \Upsilon_{\sigma_{2}}^{R_{g}} & =-\frac{\sigma_{2}(\mu +\sigma_{1})}{\mu (\gamma +\mu +\sigma_{2})+\sigma_{1}(\gamma \varepsilon +\mu +\sigma_{2})},\\ \Upsilon_{\theta }^{R_{g}} & =\frac{\theta \mu p}{\left(\theta +\mu )(\theta +\mu (1-p)\right)},\\ \Upsilon_{p}^{R_{g}} & =-\frac{\mu p}{\theta +\mu (1-p)},\\ \Upsilon_{\gamma }^{R_{g}} & =-\frac{\gamma (\mu +\sigma_{1}\varepsilon )}{\mu (\gamma +\mu +\sigma_{2})+\sigma_{1}(\gamma \varepsilon +\mu +\sigma_{2})},\\ \;\text{and}\;\Upsilon_{\varepsilon }^{R_{g}} & =-\frac{\gamma \sigma_{1}\varepsilon }{\mu (\gamma +\mu +\sigma_{2})+\sigma_{1}(\gamma \varepsilon +\mu +\sigma_{2})}. \end{array}$$

From the calculations here we see that $R_{g}$ is most sensitive to changes in the values of *β* and *Λ*. An increase in either of these results in an increase of the same proportion in $R_{g}$ and a decrease in either of these will bring about an equivalent decrease in the value of $R_{g}$; they are directly proportional. Also, $R_{g}$ has a direct proportional relationship with parameters *σ*_1_ and *θ*, however with a proportionally smaller increase or decrease. Parameters *σ*_2_, *p*, *γ* and *ε* have an inversely proportional relationship with $R_{g}$; an increase in any of them will bring about a decrease in $R_{g}$. This is a reflection that increasing the efficiency of correctional services by administering more restorative and corrective interventions for gang members can be of crucial help in enabling safe transition of offenders back to the community.

To further understand the model reproduction number in the context of gangs, we can deduce the threshold efficacy by setting $R_{g}=1$. It can easily be established that

$$\varepsilon^{*}=\left(\frac{\mu (\mu +\gamma +\sigma_{2})+\sigma_{1}(\mu +\sigma_{2})}{\gamma \sigma_{1}}\right)(R_{0}-1),$$

where

3.4 $$R_{0}=\frac{\beta \Lambda (\mu +\sigma_{1})(\theta +\mu (1-p))}{\mu (\theta +\mu )(\mu (\gamma +\mu +\sigma_{2})+\sigma_{1}(\mu +\sigma_{2}))},$$

is the basic reproduction number, the value of $R_{g}$ in the absence of correctional services i.e *ε*=0. In the absence of correctional services (obtained by setting *ε*=0), the model assumes that there is no rehabilitative correction and individuals released from correctional facilities go back into gangs. An efficacy of *ε*=0 depicts totally dysfunctional correctional services, while *ε*=1 signifies that correctional services will be 100% effective. A high value of the efficacy of correctional services in any given population impacts the reproduction number over time. The question then is: what is the threshold efficacy necessary for the reduction of the reproduction number to below one? Absence of correctional services here means that jails do not act as rehabilitation and correctional facilities. So gangs can be contained or eradicated if the efficacy of correctional services is maintained above ε*. This clearly shows the need to have restorative and corrective prisons for gang members. Some corrective interventions include skilling, counselling and education of inmates. Research has shown that offenders who undergo programmes such as the provision of education, employment and other correctional programmes (e.g. substance abuse, violence prevention, sexual offending prevention, family violence prevention), at the most appropriate time in the offender’s sentence, contributes to safe transition to the community. Education programmes in custodial settings are known to decrease recidivism [3].

### 3.4. Local stability of the gang-free steady state

We shall now prove the local stability of the gang-free equilibrium point $G^{0}$ whenever the gang reproduction number $R_{g}$ is less than unity.

**Theorem 3.2**

*The gang-free equilibrium point* $G^{0}$ *is locally asymptotically stable if* $R_{g}<1$ *and unstable otherwise.*

**Proof.**

The Jacobian matrix evaluated at $G^{0}$ is

$$J(G^{0})=\left(\begin{array}{cccc} -\theta -\mu & 0 & 0 & \varepsilon \nu \sigma_{1}\\ \theta & -\mu & \sigma_{2}-\beta S_{r}^{0} & \varepsilon (1-\nu )\sigma_{1}\\ 0 & 0 & \beta S_{r}^{0}-(\mu +\gamma +\sigma_{2}) & (1-\varepsilon )\sigma_{1}\\ 0 & 0 & \gamma & -(\mu +\sigma_{1}) \end{array}\right).$$

The eigenvalues are given by *λ*_1_=−(*μ*+*θ*), *λ*_2_=−*μ* and the solution of

$$\left|\begin{array}{cc} \beta S_{r}^{0}-Q_{1}-\lambda & (1-\varepsilon )\sigma_{1}\\ \gamma & -Q_{2}-\lambda \end{array}\right|=0.$$

This gives

$$\lambda^{2}+(Q_{1}+Q_{2}-\beta S_{r}^{0})\lambda +(\mu Q_{2}+\sigma Q_{3})(1-R_{g})=0,$$

where *Q*_1_=*μ*+*σ*_1_, *Q*_2_=*μ*+*γ*+*σ*_2_ and *Q*_3_=*γε*+*μ*+*σ*_2_. We note that when $R_{g}<1$, then the remaining eigenvalues will be both negative. This completes the proof. ▪

### 3.5. Gang-persistent equilibrium

In this section, we determine the gang-persistent equilibrium point denoted by $G^{*}=(S_{n}^{*},S_{r}^{*},G^{*},C^{*})$. The gang-persistent equilibrium always satisfies

3.5 $$\begin{array}{rl} 0 & =p\Lambda +\nu \varepsilon \sigma_{1}C^{*}-(\mu +\theta )S_{n}^{*},\\ 0 & =(1-p)\Lambda +\theta S_{n}^{*}+\sigma_{2}G^{*}+(1-\nu )\varepsilon \sigma_{1}C^{*}-\beta G^{*}(1+\eta G^{*})S_{r}^{*}-\mu S_{r}^{*},\\ 0 & =\beta G^{*}(1+\eta G^{*})S_{r}^{*}+(1-\varepsilon )\sigma_{1}C^{*}-(\mu +\sigma_{2}+\gamma )G^{*}\\ \;\text{and}\;0 & =\gamma G^{*}-(\mu +\sigma_{1})C^{*}. \end{array}\}$$

From the last equation of (3.5), we have that

3.6 $$C^{*}=\frac{\gamma G^{*}}{\mu +\sigma_{1}}.$$

Substituting equation (3.6) into the first and third equation of (3.5) leads to

3.7 $$S_{n}^{*}=\frac{\sigma_{1}(\gamma G^{*}\nu \varepsilon +\Lambda p)+\Lambda \mu p}{\left(\theta +\mu )(\mu +\sigma_{1}\right)}\;\text{and}\;S_{r}^{*}=\frac{\mu (\gamma +\mu +\sigma_{2})+\sigma_{1}(\gamma \varepsilon +\mu +\sigma_{2})}{\beta (\eta G^{*}+1)(\mu +\sigma_{1})}.$$

Substituting equations (3.6) and (3.7) into the second equation of (3.5) leads to the following quadratic equation in terms of G*

3.8 $$aG^{*2}+bG^{*}+c=0,$$

where

$$\begin{array}{rl} a & =-\beta \eta \mu ((\gamma +\mu )(\theta +\mu )+\sigma_{1}(\gamma \nu \varepsilon +\theta +\mu )),\\ b & =\beta (\eta \Lambda (\theta +\mu (1-p))(\mu +\sigma_{1})-\mu (\mu +\gamma )(\mu +\theta )-\mu \sigma_{1}(\gamma \nu \varepsilon +\theta +\mu ))\\ \;\mathrm{and}\;c & =\mu (\theta +\mu )(\mu (\gamma +\mu +\sigma_{2})+\sigma_{1}(\gamma \varepsilon +\mu +\sigma_{2}))(R_{g}-1). \end{array}$$

Define now the following quantities

3.9 $$\begin{array}{rl} \eta^{*} & =\frac{\mu (\mu +\gamma )(\mu +\theta )+\mu \sigma_{1}(\gamma \nu \varepsilon +\theta +\mu )}{\Lambda (\theta +\mu (1-p))(\mu +\sigma_{1})}\\ \;\mathrm{and}\;R_{g}^{*} & =\frac{\beta {\left(\eta \Lambda (\theta +\mu (1-p))(\mu +\sigma_{1})-\mu (\mu +\gamma )(\mu +\theta )-\mu \sigma_{1}(\gamma \nu \varepsilon +\theta +\mu )\right)}^{2}}{4\mu^{2}\eta (\mu +\theta )((\gamma +\mu )(\theta +\mu )+\sigma_{1}(\gamma \nu \varepsilon +\theta +\mu ))(\mu (\gamma +\mu +\sigma_{2})+\sigma_{1}(\gamma \varepsilon +\mu +\sigma_{2}))}. \end{array}\}$$

For the gang-persistent equilibrium to exist, the solutions of (3.8) must be real and positive. We note

(*G*1) *a*<0,
(*G*2) *b*≤0⇔*η*≤η* and *b*>0⇔*η*>η*,
(*G*3) $c\leq 0\Leftrightarrow R_{g}\leq 1$ and $c>0\Leftrightarrow R_{g}>1$.

Since *a*≠0, equation (3.8) is a quadratic equation with respect to G*. Let the discriminant of (3.8) be denoted by *Δ*, so that

3.10 $$\Delta =R_{g}^{*}+R_{g}-1.$$

Solving (3.10) for *Δ*=0 in terms of $R_{g}$, we get

3.11 $$R_{g}=1-R_{g}^{*}.$$

We clearly note the following relations:

$$\Delta >0\;⟺\;R_{g}>1-R_{g}^{*},\;\Delta <0\;⟺\;R_{g}<1-R_{g}^{*}.$$

Equation (3.8) has real roots provided *Δ*≥0. We thus have the following results on existence of the gang-persistent equilibrium.

**Theorem 3.3**

*The following results hold.*

(H1) *Let η*=0. *Then, system* (2.1) *has a unique gang-persistent equilibrium when* $R_{g}>1$.
(H2) *Let η*>0; *system* (2.1) *has*
  (i) a unique gang-persistent equilibrium when η>η* *and* $R_{g}>1$*;*
  (ii) a unique gang-persistent equilibrium when η<η* *and* $R_{g}\geq 1$*;*
  (iii) *two gang-persistent equilibria* $G_{1}^{*}=(S_{n1}^{*},S_{r1}^{*},G_{1}^{*},C_{1}^{*})$ *and* $G_{2}^{*}=(S_{n2}^{*},S_{r2}^{*},G_{2}^{*},C_{2}^{*})$ when η>η* *and* $1-R_{g}^{*}<R_{g}<1$ *where* $G_{1}^{*}=\left(-b+\sqrt{b^{2}-4ac}\right)/2a$ *and* $G_{2}^{*}=\left(-b-\sqrt{b^{2}-4ac}\right)/2a$*;*
  (iv) no gang-persistent equilibria when η>η* *and* $R_{g}<1-R_{g}^{*}$*; and*
  (v) no gang-persistent equilibria when η<η* *and* $R_{g}<1$.

Epidemiologically, a backward bifurcation entails that it is not enough to only reduce the basic reproductive number to less than 1 to eliminate a disease. On most part there are two distinct bifurcations at $R_{0}=1$ namely; forward (supercritical) and backward (subcritical). A backward bifurcation happens when $R_{0}$ is less than unity, a small positive unstable equilibrium appears while the disease-free equilibrium and a larger positive equilibrium are locally asymptotically stable. On the other hand, a forward bifurcation happens when $R_{0}$ crosses unity from below, a small positive asymptotically stable equilibrium appears and the disease-free equilibrium looses its stability [31]. The phenomenon of backward bifurcation was first found in epidemiological models by Huang *et al.* [32]. Studies supporting backward bifurcations include those in [33–37].

From theorem 3.3, we observe that if *η*=0, then system (2.1) has a unique gang-persistent equilibrium when $R_{g}>1$. For this case, the bifurcation at $R_{g}=1$ is forward. However, if *η*>0, system (2.1) has two different gang-persistent equilibria when *η*>η* and $1-R_{g}^{*}<R_{g}<1$. Hence, system (2.1) has a backward bifurcation at $R_{g}=1$ from the gang-free equilibrium to two gang-persistent equilibria. To conclude, we now show existence of backward bifurcation.

### 3.6. Backward bifurcation

Conditions for the existence of backward bifurcation follow from Theorem 4.1 proved in [31]. We deliberately avoid rewriting the theorem and focus on its application. Let us make the following change of variables: *S*_*n*_=*x*_1_, *S*_*r*_=*x*_2_, *G*=*x*_3_, *C*=*x*_4_, so that $N=\sum_{n=1}^{4}x_{n}$. We now use the vector notation *X*=(*x*_1_,*x*_2_,*x*_3_,*x*_4_)^T^. Then, model system (2.1) can be written in the form d*X*/d*t*=*F*(*t*,*x*(*t*))=( *f*_1_,*f*_2_,*f*_3_,*f*_4_)^T^, where

3.12 $$\begin{array}{rl} \frac{dx_{1}}{dt} & =p\Lambda +\nu \varepsilon \sigma_{1}x_{4}-(\mu +\theta )x_{1}=f_{1},\\ \frac{dx_{2}}{dt} & =(1-p)\Lambda +\theta x_{1}+\sigma_{2}x_{3}+(1-\nu )\varepsilon \sigma_{1}x_{4}-\beta x_{3}(1+\eta x_{3})x_{2}-\mu x_{2}=f_{2},\\ \frac{dx_{3}}{dt} & =\beta x_{3}(1+\eta x_{3})x_{2}+(1-\varepsilon )\sigma_{1}x_{4}-(\mu +\sigma_{2}+\gamma )x_{3}=f_{3}\\ \;\mathrm{and}\;\frac{dx_{4}}{dt} & =\gamma x_{3}-(\mu +\sigma_{1})x_{4}=f_{4}. \end{array}\}$$

Let *β* be the bifurcation parameter, $R_{g}=1$ corresponds to

3.13 $$\beta =\beta^{*}=\frac{\mu (\theta +\mu )(\mu (\gamma +\mu +\sigma_{2})+\sigma_{1}(\gamma \varepsilon +\mu +\sigma_{2}))}{\Lambda (\mu +\sigma_{1})(\theta +\mu (1-p))}.$$

The Jacobian matrix of model system (2.1) at $G^{0}$ when *β*=β* is given by

$$J^{*}(G^{0})=\left(\begin{array}{cccc} -(\mu +\theta ) & 0 & 0 & \varepsilon \nu \sigma_{1}\\ \theta & -\mu & \sigma_{2}-\frac{\beta^{*}\Lambda (\theta +(1-p)\mu )}{\mu (\theta +\mu )} & \varepsilon (1-\nu )\sigma_{1}\\ 0 & 0 & \frac{\beta^{*}\Lambda (\theta +(1-p)\mu )}{\mu (\theta +\mu )}-(\mu +\gamma +\sigma_{2}) & (1-\varepsilon )\sigma_{1}\\ 0 & 0 & \gamma & -(\mu +\sigma_{1}). \end{array}\right)$$

Model system (3.12), with *β*=β* has a simple eigenvalue, hence the centre manifold theory can be used to analyse the dynamics of model system (2.1) near *β*=β*. It can be shown that $J^{*}(G^{0})$, has a right eigenvector given by *w*=(*w*_1_,*w*_2_,*w*_3_,*w*_4_)^T^, where

3.14 $$\begin{array}{lll} & w_{1}=\gamma \nu \sigma_{1}\varepsilon , & w_{2}=-(\gamma +\mu )(\theta +\mu )-\sigma_{1}(\gamma \nu \varepsilon +\theta +\mu ),\\ \;\mathrm{and}\; & w_{3}=(\theta +\mu )(\mu +\sigma_{1}), & w_{4}=\gamma (\theta +\mu ). \end{array}\}$$

Further, the left eigenvector of $J^{*}(G^{0})$, associated with the zero eigenvalue at *β*=β* is given by *v*=(*v*_1_,*v*_2_,*v*_3_,*v*_4_)^T^, where

3.15 $$v_{1}=v_{2}=0,\;v_{3}=\mu +\sigma_{1},\;v_{4}=(1-\varepsilon )\sigma_{1}.$$

The computations of **a** and **b** are necessary in order to apply Theorem 4.1 in [31]. For system (3.12), the associated non-zero partial derivatives of *F* at the gang-free equilibrium are as follows:

$$\begin{array}{rl} \frac{\partial^{2}f_{2}}{\partial x_{2}\partial x_{3}} & =\frac{\partial^{2}f_{2}}{\partial x_{3}\partial x_{2}}=-\beta^{*},\;\frac{\partial^{2}f_{2}}{\partial x_{3}^{2}}=\frac{-2\eta \Lambda \beta^{*}(\theta +(1-p)\mu )}{\mu (\mu +\theta )},\\ \frac{\partial^{2}f_{3}}{\partial x_{2}\partial x_{3}} & =\frac{\partial^{2}f_{2}}{\partial x_{3}\partial x_{2}}=\beta^{*},\;\frac{\partial^{2}f_{3}}{\partial x_{3}^{2}}=\frac{2\eta \Lambda \beta^{*}(\theta +(1-p)\mu )}{\mu (\mu +\theta )},\\ \frac{\partial^{2}f_{2}}{\partial x_{3}\partial \beta^{*}} & =\frac{-\Lambda (\theta +(1-p)\mu )}{\mu (\mu +\theta )},\;\frac{\partial^{2}f_{3}}{\partial x_{3}\partial \beta^{*}}=\frac{\Lambda (\theta +(1-p)\mu )}{\mu (\mu +\theta )}. \end{array}$$

It thus follows that

$$\begin{array}{rl} \text{a} & =v_{3}w_{2}w_{3}\frac{\partial^{2}f_{3}}{\partial x_{2}\partial x_{3}}+v_{3}w_{3}w_{2}\frac{\partial^{2}f_{3}}{\partial x_{3}\partial x_{2}}+v_{3}w_{3}^{2}\frac{\partial^{2}f_{3}}{\partial x_{3}^{2}}\\ & =\frac{2\Lambda \beta^{*}}{\mu }(\mu +\theta )(\theta +(1-p)\mu ){\left(\mu +\sigma_{1}\right)}^{3}(\eta -\eta^{*}), \end{array}$$

where η* is given in equation (3.9). Note that if *η*>η* then **a**>0 and **a**<0 if *η*<η*. Lastly,

$$\text{b}=\frac{\Lambda (\mu +\sigma_{1}){}^{2}(\theta +\mu (1-p))}{\mu }>0.$$

We thus have the following result.

**Theorem 3.4**

If η>η*, then model system (2.1*) has a backward bifurcation at* $R_{g}=1$.

**Figure 2.**
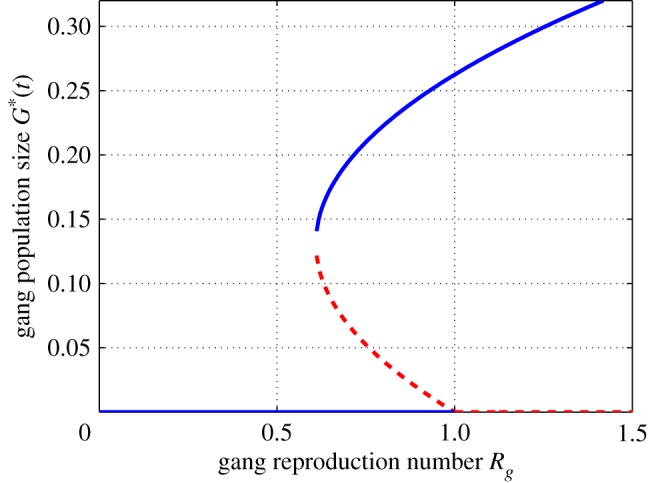
The figure showing a backward bifurcation. The *solid lines* denote stable states and the *dotted lines* denote unstable states.

From the results obtained above, we note that a backward bifurcation occurs at $R_{g}=1$ if and only if *η*>η* is satisfied. From this, we can deduce that when the imitation coefficient, *η* exceeds the critical threshold η*, then the gang population remains high leading to a backward bifurcation (figure 2). We show the existence of a backward bifurcation through numerical example by creating a bifurcation diagram around $R_{g}=1$ (figure 2). To draw a bifurcation curve (the graph of G* as a function of $R_{g}$), we fix *Λ*=0.047; *μ*=0.02; *β*=0.3; *η*=10.0; *p*=0.4; *ν*=0.7; *θ*=0.13; *γ*=0.5; *σ*_1_=0.1; *σ*_2_=0.5; *ε*=0.5. For this case, we have that η*=2.7595 and $R_{g}=0.8823$. Generally speaking, in many epidemic models the basic reproduction number, $R_{0}$, which is the key concept in epidemiology can be decreased below unity to eradicate the disease. However, in our model, this classical $R_{0}$-threshold is not the key to control the spread of gangs within a population. In fact, the existence of backward bifurcation entails that gangs may persist in the population even with values of $R_{g}$ less than unity. Our findings suggest that keeping the imitation coefficient *η* below a certain threshold η* is an effective way to avoid backward bifurcation.

## 4. Numerical simulations and results

The estimation of parameters in any model validation process is a challenging task. We make some hypothetical assumptions for the purpose of illustrating the usefulness of our model in tracking the dynamics of gangs passing through correctional services. Demographic parameters are the easiest to estimate in this instance. For the *per capita* death rate *μ*, we assume that the life expectancy of the human population is 60 years. This value has been the approximation of the life expectancy in South Africa [38]. This translates into *μ*=0.0166 per year. The recruitment of individuals in the community is linked to the birth rate. The birth rate of South Africa is on average 0.028 [39]. We thus choose a value for *Λ*=0.028. The parameters *ε*, *ν* and *p* all lie in the interval (0,1). The remaining parameters are estimated since most of them are not available in the literature and are given in table 1.

**Figure 3.**
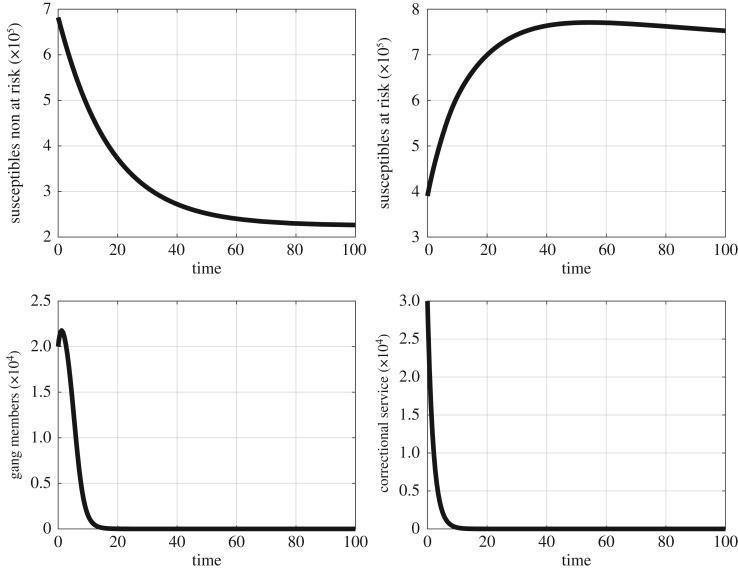
Thetime series plot showing the state variables at the gang free equilibrium, where the time is in months.

We begin by illustrating the analytic results in which the gang-free equilibrium $G^{0}$ is locally asymptotically stable when $R_{g}<1$. The results are illustrated in figure 3.

**Figure 4.**
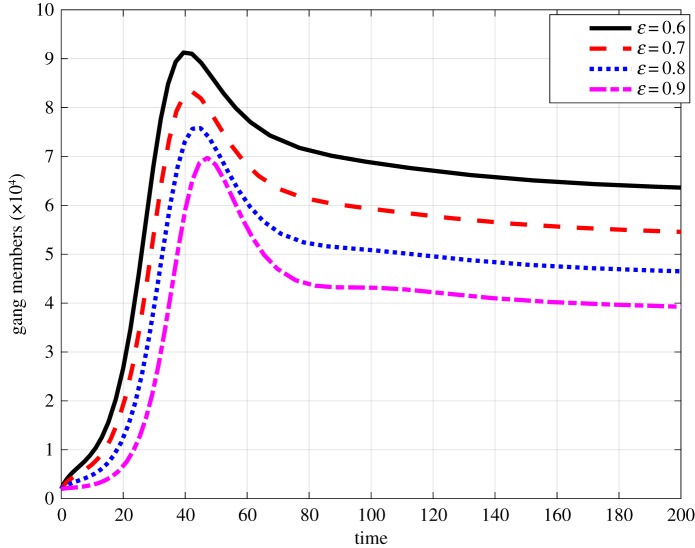
Impactof varying of *ε* on the prevalence of gang members where the time is in months.

**Figure 5.**
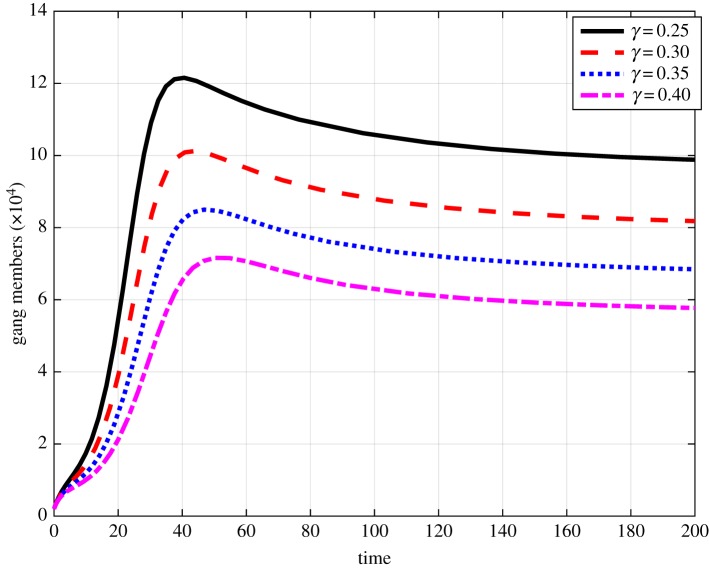
Impact ofvarying of *γ* on the prevalence of gang members where the time is in months.

We investigate the impact of the efficacy parameter *ε* on the population levels of gang members. Figure 4 shows the effects of increasing *ε* on the number of gang members. We hypothetically start at *ε*=0.6 and observe that increasing *ε* lowers the number of gang members. One can quantify the percentage decrease in the number of gang members when *ε* is increased by 0.1. For instance, an increase of *ε* for 0.6 to 0.7 reduces the number of gang members by approximately 14%. Of particular importance in the fight against gangsterism is the number of convictions on committed crimes that results in the placement of gang members in correctional services. This has been an issue of considerable concern in South Africa given the existing large variation between the number of committed crimes and the number of convictions, with conviction rates being as low as 10%. In figure 5, we begin by hypothetically setting *γ*=0.25 and observe that increasing conviction rates with a functional correctional system can lead to a reduction in the number of gang members. We observe that an increase of *γ* from 0.25 to 0.3 results in an approximate decrease of gang members by 17%.

**Figure 6.**
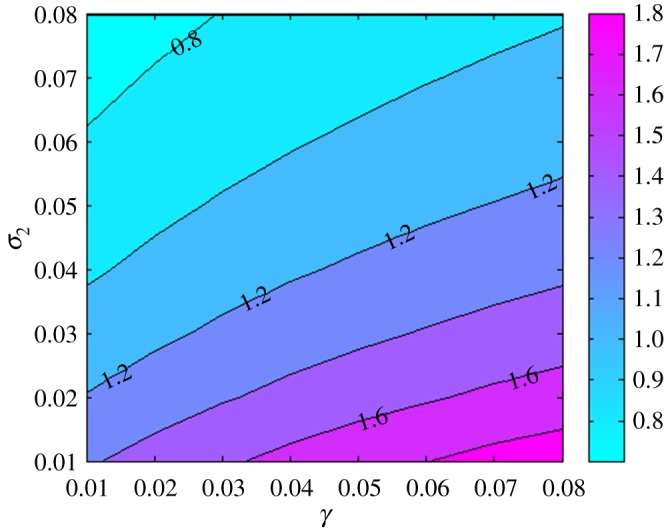
A contour plot to show how parameters *σ*_2_ and *γ* affect $R_{g}$.

Once an individual belongs to a gang, one has a choice of remaining a gang member and risk arrest as a result of gang related crimes or quitting all together. To investigate the choice a gang member has to undertake, we make a contour plot (figure 6) to show how the parameters *σ*_2_ (the rate of voluntary quitting of gang member) and *γ* (the rate of convictions and placement in correctional services) affect $R_{g}$. The results show that increasing *σ*_2_ coupled with decreasing *γ* leads to a decrease in $R_{g}$. This is of particular importance as it alludes to interventions that are not correctional. In fact, if one quits being a gang member before a conviction, that is, before crimes are committed, then its beneficial to the individual and the community since resources allocated to correctional services are saved.

## 5. Conclusion

We developed a simple mathematical model to investigate the role of correctional services on gangs. Principles drawn from the literature of mathematical epidemiology have been used to model how individuals are recruited into gangs and their possible recovery. Initiation into gangs has been assumed to happen through an imitation process in which peer influence is central to joining gangs. The growth and decrease of gang members was driven by the gang reproduction number, $R_{g}$, as in the case of epidemic models. However, in our model, this classical $R_{g}$-threshold is not the key to control gangs within communities. In fact gangs may persist in the population even with subthreshold values of $R_{g}$. It was shown to happen, in particular when the value of the imitation coefficient is high enough such that the relation *η*>η* is satisfied. In the absence of the imitation coefficient, that is, when *η*=0, the model in this study will have a unique gang-persistent equilibrium. However, the introduction of the imitation coefficient leads to multiple equilibria and seem to be responsible for interesting dynamical aspects such as the occurrence of a backward bifurcation. This means gangs may persist in the population even with subthreshold values of $R_{g}$. Thus, awareness programmes and/or specific health programmes may be employed to reduce *η* or, at least, to increase the value of η*. Our results put into evidence the importance to identify those social processes, as the imitation mechanism, that may facilitate or counteract the spread of gangs within a community of individuals. Some precise knowledge of these mechanisms is in fact essential to develop effective policies that will impede the spread of gangs within a community.

Sensitivity analysis have been performed by evaluating the sensitivity indices of the gang reproduction number, $R_{g}$, to model parameters. Since $R_{g}$ is a measure of initial gang membership, these sensitivity indices allow us to determine the relative importance of different parameters in gang initiation. It was observed that $R_{g}$ has a direct proportional relationship with the parameters *β*, *Λ*, *σ*_1_ and *θ*, whereas parameters *σ*_2_, *p*, *γ* and *ε* have an inversely proportional relationship with $R_{g}$. To further understand the role of correctional services on gangs, the threshold efficacy ε* was established. It was observed that gangs can be contained or eradicated if the efficacy of correctional services is maintained above ε*. Thus, it is important to have efficient correctional services in the fight against gangs. We also investigated the role of the efficacy parameter together with other important parameters such as conviction rates and self recovery through graphical plots.

Standard statistical techniques for collecting data on gangs such as household surveys are expensive and should, at best, be carried out every three to five years. Also, reliable gang-related data is elusive. Therefore, mathematical models become useful tools as they allow the extent of the phenomenon to be estimated. While the model presented in this study is theoretical in nature, it presents very useful and practical results that can be of help to policy makers in fighting against gangsterism, gang violence and its related crimes that have ravaged communities. Like in any model development, our model is not without limitations. The model presented in this paper assumes homogeneous mixing which is practically impossible in communities with gangs. In practice, susceptibility varies. This is because of differences in behavioural, social and environmental factors. An individual-based model could be used to address this problem. Stochastic effects can also be used to model the unpredictability of human behaviour. So inclusion of stochasticity in human behaviour can significantly improve this model. The recruitment of gang members is assumed to be driven by imitation. While this is the major initiation or recruitment driver, there is need to consider self initiation into gangs as a result of forcing circumstances, in particular, poverty. Despite these setbacks, the model presents a unique attempt to link the dynamics of correctional services and gangs mathematically. The model can also be extended by incorporating additional interventions such as behaviour change, policing and media campaigns. Our results in the presence of credible data would play a significant role in quantifying the efficacy of correctional services. In conclusion, we note that modelling gangs and correctional services mathematically raises interesting approaches to investigating the dynamics of complex criminal activities and how they relate to efforts to curb them.

## Data accessibility

Estimation of parameters have been stated throughout the body of the paper and included in the reference section. The graphs were produced using the MATLAB software that is available from https://www.mathworks.com/products/matlab.html.
